# A Neural Network for Automated Image Quality Assessment of Optic Disc Photographs

**DOI:** 10.3390/jcm12031217

**Published:** 2023-02-03

**Authors:** Ella Bouris, Tyler Davis, Esteban Morales, Lourdes Grassi, Diana Salazar Vega, Joseph Caprioli

**Affiliations:** 1 Department of Ophthalmology, Jules Stein Eye Institute, University of California-Los Angeles, Los Angeles, CA 90095, USA; 2 Department of Computer Science, University of California-Los Angeles, Los Angeles, CA 90095, USA; 3 Department of Ophthalmology, Vision Consultants and Surgeons, Falls Church, VA 22046, USA

**Keywords:** neural network, glaucoma, fundus photograph, optic nerve, image-quality assessment, code-free deep learning

## Abstract

This study describes the development of a convolutional neural network (CNN) for automated assessment of optic disc photograph quality. Using a code-free deep learning platform, a total of 2377 optic disc photographs were used to develop a deep CNN capable of determining optic disc photograph quality. Of these, 1002 were good-quality images, 609 were acceptable-quality, and 766 were poor-quality images. The dataset was split 80/10/10 into training, validation, and test sets and balanced for quality. A ternary classification model (good, acceptable, and poor quality) and a binary model (usable, unusable) were developed. In the ternary classification system, the model had an overall accuracy of 91% and an AUC of 0.98. The model had higher predictive accuracy for images of good (93%) and poor quality (96%) than for images of acceptable quality (91%). The binary model performed with an overall accuracy of 98% and an AUC of 0.99. When validated on 292 images not included in the original training/validation/test dataset, the model’s accuracy was 85% on the three-class classification task and 97% on the binary classification task. The proposed system for automated image-quality assessment for optic disc photographs achieves high accuracy in both ternary and binary classification systems, and highlights the success achievable with a code-free platform. There is wide clinical and research potential for such a model, with potential applications ranging from integration into fundus camera software to provide immediate feedback to ophthalmic photographers, to prescreening large databases before their use in research.

## 1. Introduction

Evaluation of the optic disc is an integral part of the diagnosis and management of glaucoma, as structural damage to the optic nerve can often be detected even before visual field defects are present [1,2]. Careful recognition of preperimetric morphological changes to the optic nerve is vital in monitoring disease progression [3,4,5]. Structural assessment of the optic disc can be accomplished with optical coherence tomography (OCT) and fundus photography. Subjective evaluation of optic disc photographs by clinicians is hampered by poor interobserver agreement [6,7,8,9]. Fundus photography is appropriate for both population screening and long-term follow-up, as it can be low cost and allows for direct qualitative comparison to previously captured images, even images decades old. This stands in contrast to OCT, where high cost may impede wide adoption and rapid technological innovation makes comparison with historical images difficult. Both types of testing require training to capture images of sufficient quality for use in diagnostics.

In fundus photography, as in all medical imaging, poor image quality may confound accurate assessment of the image and render images diagnostically useless. Various studies have estimated the occurrence of poor-quality fundus photographs of the optic nerve head at 3.7–25% of all images captured, meaning that automated detection of these images has immediate potential for clinical utility [10,11,12,13]. Automated image-quality assessment (IQA) is also useful as a first “quality-control” step in the research and development of other computer vision and artificial intelligence (AI) systems.

Increasingly, AI is being explored for use in the diagnosis and management of glaucoma through automated assessment of the optic disc or fundus photographs. Models developed for this task use a variety of machine learning techniques. Statistical classifiers such as support vector machine (SVM), random forest algorithms, and k-nearest neighbor are common in earlier models; recent research has focused more heavily on the use of deep learning techniques such as convolutional neural networks [14,15]. Automated detection of glaucoma is sometimes based on explicit recognition of pathological differences in certain ocular features, such as retinal nerve fiber layer defects, presence of disc hemorrhages, extent of peripapillary atrophy, or cup-to-disc ratio [16,17,18,19,20]. Other studies have relied on more complex inputs (i.e., entire images), which allows for less supervised and more holistic classification [21,22,23]. The large-scale fundus photograph databases required for developing the latter category of models in particular would benefit from a reliable automated system for screening out unusable or poor-quality images. Furthermore, there is research demonstrating the detrimental impact of poor-quality images on the diagnostic ability of these algorithms [24,25,26].

The vast majority of prior research on automated ocular IQA has focused on retinal imaging nonspecific to glaucoma. There is minimal research looking specifically at IQA for glaucoma, which has a unique set of diagnostic criteria based on the appearance of the optic disc, which requires certain anatomical structures be clearly visible. Furthermore, to our knowledge, all research in this area has used conventional machine learning pathways, which require significant expertise and computational power. In this study, we describe the development of a deep convolutional neural network (CNN) created by using a code-free deep learning platform for automated assessment of optic disc photograph quality.

## 2. Materials and Methods

The deep convolutional neural network was developed with a collection of 2377 disc photographs randomly selected from a database of fundus images captured between 1997 and 2020 at the Glaucoma Division of the UCLA Jules Stein Eye Institute. There were no exclusion criteria applied during image selection, meaning that multiple images from the same patient or the same eye taken on different dates could potentially be included. To minimize bias, if multiple images from the same patient were included, they appear in only one of the split sets—either the training, validation, or test sets.

A total of 2377 optic disc photographs from 1684 eyes of 1360 patients at a glaucoma subspecialty clinic were used to train, validate, and test a convolutional neural network in an 80/10/10 split. Totals of 1626 digitized scanned slides and 751 digital images were used. The slide images were captured with a Zeiss Fundus Flash 3 Camera on Kodachrome 25 film and digitized at a third-party company. The digital images were captured on either a Zeiss Fundus Flash 3 with Escalon Digital Back or a Zeiss FF450 with Digital Back. All images were converted to the same image type (JPEG) when input into the model.

**Figure 1:**
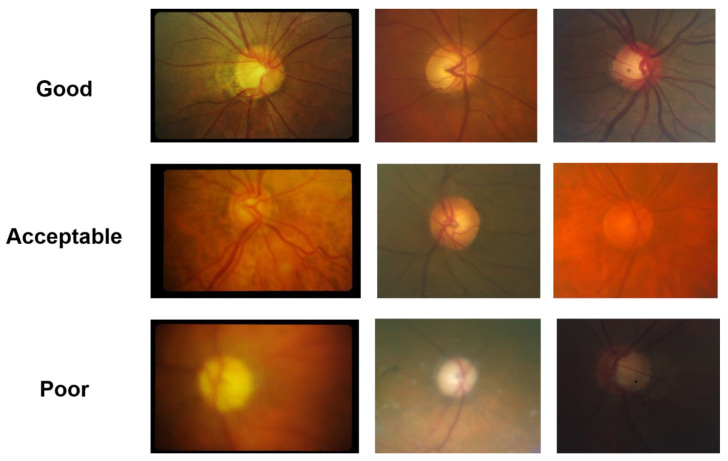
Standard reference photographs for each image quality class.

Prior to grading the images, standard reference photographs were chosen by three glaucoma specialists to define the range of image quality acceptable at each level (Figure 1). Using a web-based interface, images were sorted into three quality classes (good, acceptable, and poor) by one of three glaucoma specialists and subsequently used to develop the model. Our dataset contained 1002 good-quality images, 609 acceptable-quality images, and 766 poor-quality images.

A convolutional neural network was developed with Google Cloud AutoML, a code-free deep learning platform offered by Google. The AutoML platform allowed local data manipulation and subsequent upload of .csv files containing the cloud location of the image file and the grader-assigned image quality. The platform automates image preprocessing and the model training and selection process, including neural architecture search and hyperparameter tuning. To minimize overfitting, early stopping was enabled, which automatically terminated training when no more improvements could be made. Automated internal image preprocessing occurred when the image’s smallest edge was greater than 1024 pixels. In this case, the entire image was scaled down so the smallest edge was 1024 pixels. Images with a small side less than or equal to 1024 pixels were not subject to preprocessing.

Of the 2377 image dataset, 80% (1897/2377) were used for training the model, 10% (242/2377) were used to validate the model, and 10% (263/2377) were used to test the model’s performance. The same proportions (approximately 33% each) of good-, acceptable-, and poor-quality images were included in each dataset. The model’s output was three prediction values, one corresponding to each quality level (good, acceptable, poor), ranging from 0–1 and summing to 1. For each image, the category with the highest score was taken as the model’s predicted quality label.

We performed independent validation on a set of 292 randomly selected disc photographs that were not part of the 2377 images used to develop the model. In this round of validation, the model’s performance was measured against a consensus-derived grade from three glaucoma specialists who evaluated the images independently. Consensus was defined as agreement between two of the three graders; there were no instances of disagreement between all three clinicians.

## 3. Results

Table 1 provides a tabulation of the demographic characteristics of the patients represented in this dataset. Fifty-six percent were female, and the average age at the time of the disc photograph was 64 years old. Patients were primarily Caucasian (54.6%); Asian and Black patients constituted 14.0% and 10.7% of the study population, respectively. Among the included eyes, 47.3% had a diagnosis of primary open angle glaucoma, and 22.3% were considered suspicious for glaucoma at the time of image capture.

**Figure 2:**
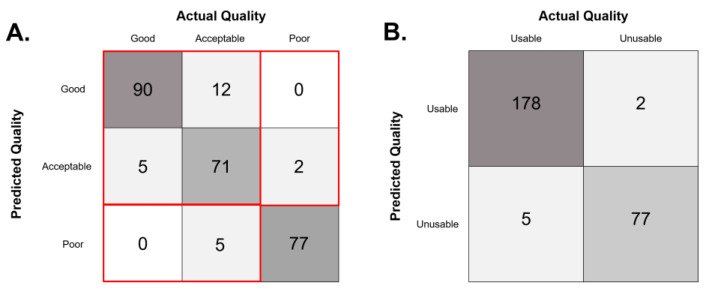
(**A**) Raw count confusion matrix for the three-class model tested on 262 glaucoma-specialist graded disc photographs. Areas outlined in red show the regions collapsed to generate the binary outcome confusion matrix represented in (**B**).

Results are presented for both the ternary classification system described in the Methods section, and the binary classification that results from combining the “good” and “acceptable” classes into one “usable” class, vs. “unusable”, corresponding to label “poor” of the three-class model. Figure 2 shows confusion matrices for both outcomes.

### 3.1. Ternary Classification Model

Full performance metrics (per-class and macro average) for the three-class model are shown in Table 2. AUC was calculated by using a one vs. rest approach, which splits the multi-classification task into several binary classification tasks, (e.g., good vs. [acceptable, poor]), then averages the AUC for each class to arrive at a single value. Macroaveraging, in which all classes get equal weight, was used because the three quality classes were relatively balanced. AUC for the model was 0.98, and the CNN had an overall accuracy of 91%. Although per-class performance varied between different metrics, generally the model performed better on classification of “good” and “poor” images than on images of “acceptable” quality.

**Figure 3:**
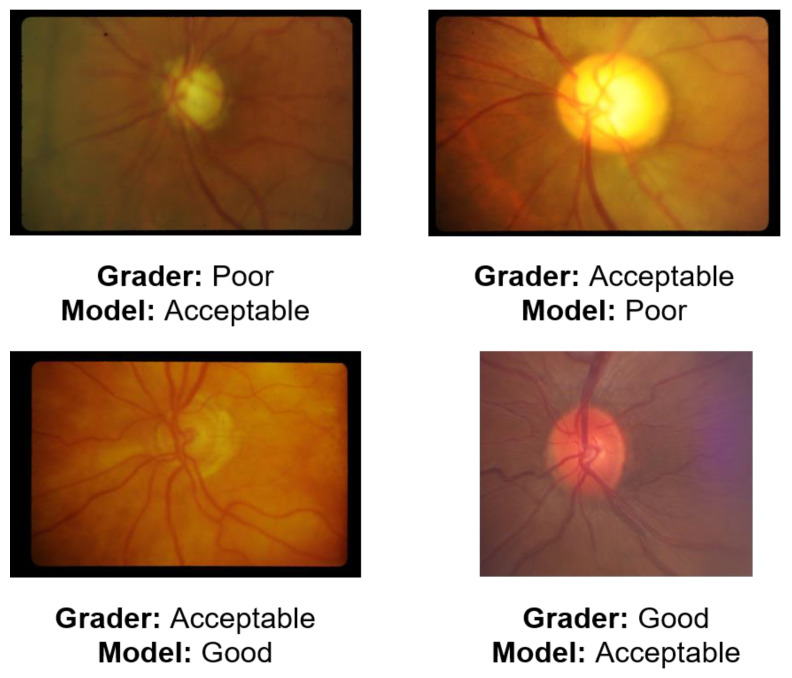
Representative sample of images mispredicted by the ternary classification model.

Examples of images misclassified by the model are shown in Figure 3. Notably, all misclassifications were no more than one category removed from their actual quality label (i.e., no “good” images mistaken for “poor” and vice versa).

Females were 33% more likely to have images of poor quality than good quality (OR 1.33, 95% CI [1.10, 1.60]). There was no significant gender difference between the good- and acceptable-quality groups. On average, age at the time of disc photograph capture was significantly higher in images of acceptable (7.45 years, adjusted *p* < 0.01) and poor (7.71 years, adjusted *p* < 0.01) quality than in those of good quality.

### 3.2. Binary Classification Model

Full classification metrics for the binary outcome model can be found in Table 3. Overall, performance was better on the binary outcome model than the three-class model, with an accuracy of 98.47%. Sensitivity and specificity were both high, at 98.91% and 97.47%, respectively. AUC was also high at 0.99. None of the collected demographic information varied significantly between the usable and unusable quality groups.

### 3.3. Independent Validation

Independent validation on 292 randomly selected images not included in the 2377-image dataset used to develop the model was performed to further evaluate the robustness of the model. Based on a three-clinician consensus grading, there were 225 good-, 53 acceptable-, and 14 poor-quality images in this dataset. This distribution is likely more representative of the entire optic disc photograph database.

The model agreed with the clinician consensus in 84.9% of cases, with a kappa value of 0.66. This corresponded to a 97.3% agreement with clinicians (kappa = 0.65) when considered as a binary outcome task, usable vs. unusable.

## 4. Discussion

The proposed system for automated IQA on optic disc fundus photographs achieves high accuracy for both the three-class task (90.84%) and the binary outcome task (98.47%), demonstrating that a CNN is able to provide a high degree of discrimination between images of good, acceptable, and poor quality. There is a wide breadth of literature concerning IQA and its utility in nonglaucoma pathologies, but there is comparatively little specific to glaucoma. We will be discussing both to contextualize our model and its performance.

### 4.1. Binary Approach—Non-Glaucomatous Fundus Photographs

In order to perform retinal IQA as a preliminary step in diabetic retinopathy screening, Saha et al. fine-tuned a pretrained AlexNet model to classify images from patients with diabetic retinopathy into the categories “accept” and “reject” [27]. AUC, sensitivity, specificity, and accuracy were all 100%. However, the authors excluded all “ambiguous” images, defined by a lack of agreement in image quality among the graders, from the training dataset. When these images were included, the model’s accuracy dropped to 54.5%, highlighting a major limitation of in the model’s potential utility in a clinical setting. Similarly, Zago et al. adapted the Inception v3 architecture to the task of fundus IQA, again for eyes with retinal pathology [28]. The model’s performance was evaluated by using interdataset cross-validation on two publicly available datasets, DRIMDB and ELSA-Brasil. The authors used image augmentation to synthesize poor-quality images, which were poorly represented in both datasets, and achieved a high sensitivity and specificity (97% and 100%, respectively, on DRIMDB; 92% and 96% on ELSA-Brasil) in the cross-validation. Another recent paper by Chalakkal et al. described a unique approach to IQA [29]. Their model first uses a deep learning classifier based only on image-quality markers such as sharpness and illumination, then performs a second round of unsupervised classification on images the initial classifier marked as good quality. Good quality in the second stage is defined by the presence of certain structural features of the retina, such as the optic disc, fovea, and macula. The authors report successful outcomes by using this approach, with an overall accuracy of 97.47%.

### 4.2. Binary Approach—Glaucomatous Optic Disc Photographs

Despite the success of the aforementioned models, they are not readily applicable to images of glaucomatous optic discs due to the domain-specific way in which image quality is defined. Diagnostically relevant pathological optic nerve head features such as vascular abnormalities, parapapillary atrophy, and disc hemorrhages can be difficult for a neural network to distinguish from poor-quality images or images with artifacts, and as such, it is important to have a model trained on images of glaucomatous discs [13,30,31,32]. Retinal IQA models traditionally focus on vessel visibility as the most important grading criterion, while IQA for imaging performed on glaucoma patients would be better served by an assessment of image quality in a region dominated by the optic disc [33]. There has been relatively little research performed specifically on this disease population, perhaps due in part to the scarcity of large, disease-specific, quality-labeled image databases [34].

Mahapatra et al. attempted to combat these gaps in large, publicly available datasets by first extracting 30,000 150 × 150 pixel patches from the 101 images in the DRISHTI glaucoma dataset, then using image augmentation (adding noise, altering contrast, etc.) to artificially generate poor-quality images, as the original dataset contained none [35]. They saw high accuracy (99.87%) by using these methods, and to the best of our knowledge were the first group to build a CNN-based IQA system specific to glaucomatous optic disc photographs. The next group to do so, Zapata et al., used 150,075 quality-labeled retinal images to train a model to discern good vs. poor image quality, with an overall accuracy of 92% [36]. It is unclear what portion of these images were from patients with glaucoma; 3776 of their total 306,302 fundus photographs were considered to have “referable glaucomatous optic neuropathy”. The authors defined good-quality images as those centered on the macula with good focus, good visualization of the parafoveal vessels and at least two disc diameters around the fovea, and ability to visualize at least three quarters of the optic disc and the vascular arcades. This definition highlights our earlier point regarding disease-specific criteria for image quality—in our study, as well as in a 2020 study by Bhatkalkar et al., visualization of the entire optic disc was necessary for the graders to consider an image of good quality [37]. The lack of a standardized metric for defining image quality makes direct comparison between models difficult, a pervasive theme in the domain of IQA. Bhatkalkar’s model, which was trained by using three publicly available image databases along with their own hospital database and was tested on three small public datasets, had an accuracy ranging from 96.4% to 100%. Notably, their model was trained on fundus photograph datasets of patients with diabetic retinopathy and age-related macular degeneration, and thus is not generalizable to the assessment of IQA on patients with glaucomatous damage to the optic nerve. However, it does provide an indication of the success that may be achievable in such populations.

Our model achieved performance similar to that reported for other IQA systems. As all the reported glaucoma-specific IQA systems provide binary outputs, we will be directly comparing the performance of our binary model. Our binary classification model shows comparable accuracy to the models developed by Mahapatra, Zapata, and Bhatkalkar (98.5% vs. 99.87%, 92%, and 96.4–100%, respectively). We report sufficiently high sensitivity (98.9) and specificity (97.5) values which are on par with those reported by Mahapatra (100, 99.8) and Zapata (96.9, 81.8). These results are also consistent with the high level of success achieved by other groups developing similar models for IQA of patients afflicted with diabetic retinopathy, age-related macular degeneration, and other retinal pathologies.

Our study is unique for several reasons, most notably the size of our training dataset and the balanced sample sizes of the three quality classes. We trained, tested, and validated the model by using a total of 2377 images, which to the best of our knowledge is the largest dataset of quality-labeled glaucomatous disc photographs used in the development of such a model. Due to the scarcity of large databases of images from glaucomatous eyes, other studies have used smaller datasets, ranging in size from 99 to 397 images [35,37]. Moreover, the majority of publicly available datasets contain insufficient proportions of poor-quality images, which can result in lower predictive accuracy for the underrepresented class. As an extreme example, in the dataset used by Saha et al., only 143 out of the total 3577 images (4%) were of “reject” quality; given this distribution, a naïve model that only outputs “accept” will achieve 96% accuracy [27]. Some groups relied on image augmentation to artificially generate poor quality images and overcome this limitation, the success of which may not be successfully transferred to clinical practice [35]. Our model’s performance also highlights the success achievable on a code-free machine learning platform, which can significantly reduce the in-house computational power and deep-learning expertise that is typically required when developing a neural network [38].

### 4.3. Multiclass Approach

Although binary classification models like those previously described make up the majority of current IQA systems, recent literature has suggested that there may be a need for a more granular classification system with more than two levels of quality. We agree that a multiclass model addresses one of the primary limitations of a binary model, namely that it has difficulty classifying images of borderline quality [13]. By denoting an intermediate “acceptable” category, we attempted to keep such images from being mixed with the highest-quality images. There is a small amount of existing literature that relies on a multilevel grading system similar to our proposed three-class methodology. In a 2019 study by Fu et al., the authors define three levels of image quality: “good” (high-quality images with all retinopathy characteristics visible), “usable” (low contrast, blur, poor illumination, or artifacts, but main structures still identifiable by ophthalmologists), and “reject” (unreliable diagnostically) [25]. They report comparable accuracy (91.75%) to our ternary classification model. Other researchers have suggested parsing out image-quality categories even further to increase clinical utility—for example, Wang et al. define five levels of quality (adequate, just noticeable blur, incomplete optic disc, under/over-illumination, and opacity) [39]. By using a 121-layer, 4 block DenseNet architecture, they applied transfer learning and fine-tuned the model to suit their five image-quality classes. Their model was relatively accurate (92.7% overall accuracy with per-class accuracy 77–100%) in discrimination between the four image degradation categories, but the wide range in per-class accuracy illustrates the challenges faced when shifting from binary to multiclass classification systems. The authors saw better outcomes in their binary classification model (good vs. poor, accuracy 97.2% and 98.2% on the two datasets used). We saw this reflected in our model’s performance as well, with overall accuracy increasing when we adapted our three-level classification to a binary system.

Although our model demonstrated a relatively high level of accuracy in both the binary and multiclass forms, it is not without limitations. As previously mentioned, binary models have difficulty classifying images of borderline quality. This is not unique to our model, nor is it unique to neural networks. Image quality, even when precisely defined, is a subjective measure on which even trained ophthalmologists often disagree [27]. We attempted to address this with a three-class model in the hopes of isolating the borderline quality images, which investigators can choose to use or discard, depending on the task at hand. Additionally, for our model to be more useful in widespread population screening, it should be tested on images captured from a wider variety of cameras, particularly nonmydriatic fundus cameras, portable cameras, and ophthalmic lens attachments for smartphones. Additional validation, particularly on an external dataset, would better assess the robustness and generalizability of our model. However, there is a relative lack of public datasets of glaucomatous optic disc photographs, and an absolute lack of quality-labeled datasets, both of which limit our ability to externally evaluate the model’s performance [34].

There is immediate potential for clinical and research utility of an IQA CNN such as ours. It has been suggested that models such as the one described in this paper can be integrated with the software of the camera capturing the fundus photographs to provide real-time feedback to photographers and allow for timely recapture of any unusable images [13]. Such an integration has the potential to improve clinical efficiency and is increasingly relevant with the advent of telemedicine in ophthalmology, where a trained ophthalmologist might not immediately be evaluating the images.

Although binary models may be more immediately applicable to clinical practice as they provide a simple outcome of diagnostically usable or unusable, a multiclass model may be better suited to certain tasks. For example, when examining serial disc photographs, image artifacts (media opacities, dust on the camera lens, patient eyelashes, lens flare, etc.) presumably present at a higher rate in the “acceptable” category may hinder accurate detection of real glaucomatous progression. Furthermore, as artificial intelligence for diagnostics and screening is increasingly the subject of ophthalmologic research, the development of large-scale fundus photograph databases is of paramount importance. Many studies have demonstrated that inclusion of low-quality images greatly decreases the accuracy of these diagnostic algorithms and limits their deployability into clinical practice [26]. A multiclass IQA network would directly benefit research efforts by allowing researchers to quickly select only appropriate quality images for use in training diagnostic models.

Future research should focus on further improving both binary and multiclass classification of optic disc IQA. Alternatively, the development of a model that outputs a continuous quality grade could allow even further control when selecting a threshold for inclusion as an image of “good” quality, offering the greatest flexibility for project-specific quality needs. Additionally, the development of a system capable of outputting the reason for assigning a “poor” quality grade (e.g., optic disc not completely in view, poor contrast, not focused, media opacity, etc.) could further enhance the clinical utility of an IQA algorithm and allow photographers to adjust their technique in real time.

## 5. Conclusions

Image quality is an important consideration when automating the process of disease diagnosis, which is a growing focus of glaucoma research. Poor-quality images are often excluded from datasets used to train machine learning models, a highly time-consuming process when the dataset size is in the order of hundreds of thousands. Moreover, when these diagnostic models are ultimately implemented into clinical practice, the presence of poor-quality images may limit their ability to correctly identify the presence of glaucomatous damage to the optic disc. Thus, a tool such as the proposed IQA model has clear applications to the development and implementation of AI-supported glaucoma diagnosis.

The proposed method classifies an optic disc photograph into one of three classes based on image quality: good, acceptable, and poor. We report outcomes for this multi-class model as well as a binary classification system where images of “good” and “acceptable” quality are all considered part of a “usable” image class. Our model, developed on a code-free deep learning platform, is highly accurate, agreeing with human graders 90.8% (three-class model) and 98.5% (binary model) of the time. These rates are comparable to several existing methods for fundus IQA and provide evidence not only for the feasibility and utility of automated IQA of glaucomatous disc photographs, but also for the effectiveness of a code-free platform usable by nonexperts for developing neural networks.

## Figures and Tables

**Table 1:** Demographic and clinical characteristics of the study population.

Variable	*n* (%)
No. of eyes (patients)	1684 (1361)
Age in years	
Mean (± standard deviation, SD)	65.0 (±16.4)
Range	5.1–97.8
Median (± interquartile range, IQR)	67.1 (±18.7)
Sex	
Female	763 (56.1%)
Male	598 (43.9%)
Race	
White	743 (54.6%)
Asian	191 (14%)
Black	145 (10.7%)
Hispanic	107 (7.9%)
American Indian or Alaska Native	9 (0.7%)
Unknown	166 (12.2%)
Eye laterality	
Right	937 (55.6%)
Left	747 (44.4%)
Glaucoma diagnosis	
Primary open angle	797 (47.3%)
Glaucoma suspect	375 (22.3%)
Primary angle closure	88 (5.2%)
Uveitic	40 (2.4%)
Congenital	12 (0.7%)
Neovascular	12 (0.7%)
Pseudoexfoliation	58 (3.4%)
Angle recession	8 (0.5%)
Steroid-induced	5 (0.3%)
Secondary open angle	45 (2.7%)
Secondary angle closure	11 (0.7%)
Anatomical narrow angles	6 (0.3%)
Low tension	23 (1.4%)
Pigmentary	16 (1.0%)
Unspecified	187 (11.1%)

**Table 2:** Per-class and macroaverage performance metrics for the ternary classification model.

	Good Quality	Acceptable Quality	Poor Quality	Macro Average
Sensitivity	0.88	0.91	0.94	0.91
Specificity	0.97	0.91	0.99	0.96
Precision	0.95	0.81	0.97	0.91
F1	0.91	0.86	0.96	0.91
Balanced Accuracy	0.93	0.91	0.96	0.93
Overall Accuracy	0.91			
Area under the receiver operating curve (AUC)	0.98			

**Table 3:** Performance metrics for the binary outcome model.

Sensitivity	0.99
Specificity	0.97
Precision	0.99
F1	0.99
Balanced Accuracy	0.98
Overall Accuracy	0.98
AUC	0.99

## Data Availability

Data sharing not applicable. No new data were created or analyzed in this study.

## References

[1] Johnson C.A., Sample P.A., Zangwill L.M., Vasile C.G., Cioffi G.A., Liebmann J.R., Weinreb R.N. (2003). Structure and Function Evaluation (SAFE): II. Comparison of Optic Disk and Visual Field Characteristics. Am. J. Ophthalmol..

[2] Tuulonen A., Airaksinen P.J. (1991). Initial Glaucomatous Optic Disk and Retinal Nerve Fiber Layer Abnormalities and Their Progression. Am. J. Ophthalmol..

[3] Medeiros F.A., Zangwill L.M., Bowd C., Sample P.A., Weinreb R.N. (2005). Use of Progressive Glaucomatous Optic Disk Change as the Reference Standard for Evaluation of Diagnostic Tests in Glaucoma. Am. J. Ophthalmol..

[4] Bowd C., Zangwill L.M., Medeiros F.A., Hao J., Chan K., Lee T.-W., Sejnowski T.J., Goldbaum M.H., Sample P.A., Crowston J.G. (2004). Confocal Scanning Laser Ophthalmoscopy Classifiers and Stereophotograph Evaluation for Prediction of Visual Field Abnormalities in Glaucoma-Suspect Eyes. Investig. Ophthalmol. Vis. Sci..

[5] Ugurlu S., Hoffman D., Garway-Heath D.F., Caprioli J. (2000). Relationship between Structural Abnormalities and Short-Wavelength Perimetric Defects in Eyes at Risk of Glaucoma. Am. J. Ophthalmol..

[6] Lichter P.R. (1976). Variability of Expert Observers in Evaluating the Optic Disc. Trans. Am. Ophthalmol. Soc..

[7] Blumberg D.M., De Moraes C.G., Liebmann J.M., Garg R., Chen C., Theventhiran A., Hood D.C. (2016). Technology and the Glaucoma Suspect. Investig. Ophthalmol. Vis. Sci..

[8] Tielsch J.M., Katz J., Quigley H.A., Miller N.R., Sommer A. (1988). Intraobserver and Interobserver Agreement in Measurement of Optic Disc Characteristics. Ophthalmology.

[9] Jampel H.D., Friedman D., Quigley H., Vitale S., Miller R., Knezevich F., Ding Y. (2009). Agreement among Glaucoma Specialists in Assessing Progressive Disc Changes from Photographs in Open-Angle Glaucoma Patients. Am. J. Ophthalmol..

[10] Boucher M.C., Gresset J.A., Angioi K., Olivier S. (2003). Effectiveness and Safety of Screening for Diabetic Retinopathy with Two NonmYdriatic Digital Images Compared with the Seven Standard Stereoscopic Photographic Fields. Can. J. Ophthalmol..

[11] Herbert H.M., Jordan K., Flanagan D.W. (2003). Is Screening with Digital Imaging Using One Retinal View Adequate?. Eye.

[12] Scanlon P.H., Malhotra R., Thomas G., Foy C., Kirkpatrick J.N., Lewis-Barned N., Harney B., Aldington S.J. (2003). The Effectiveness of Screening for Diabetic Retinopathy by Digital Imaging Photography and Technician Ophthalmoscopy. Diabet. Med. J. Br. Diabet. Assoc..

[13] Chan E.J.J., Najjar R.P., Tang Z., Milea D. (2021). Deep Learning for Retinal Image Quality Assessment of Optic Nerve Head Disorders. Asia-Pac. J. Ophthalmol..

[14] Artificial Intelligence in Glaucoma: Current Opinion in Ophthalmology. https://journals.lww.com/co-ophthalmology/Fulltext/2019/03000/Artificial_intelligence_in_glaucoma.5.aspx.

[15] Glaucoma Management in the Era of Artificial Intelligence. British Journal of Ophthalmology. https://bjo.bmj.com/content/104/3/301.long.

[16] Joshi G.D., Sivaswamy J., Prashanth R., Krishnadas S.R., Campilho A., Kamel M. (2012). Detection of Peri-Papillary Atrophy and RNFL Defect from Retinal Images. Proceedings of the Image Analysis and Recognition.

[17] Odstrcilik J., Kolar R., Tornow R.-P., Jan J., Budai A., Mayer M., Vodakova M., Laemmer R., Lamos M., Kuna Z. (2014). Thickness Related Textural Properties of Retinal Nerve Fiber Layer in Color Fundus Images. Comput. Med. Imaging Graph..

[18] Cheng J., Liu J., Xu Y., Yin F., Wong D.W.K., Tan N.-M., Tao D., Cheng C.-Y., Aung T., Wong T.Y. (2013). Superpixel Classification Based Optic Disc and Optic Cup Segmentation for Glaucoma Screening. IEEE Trans. Med. Imaging.

[19] Al-Bander B., Williams B.M., Al-Nuaimy W., Al-Taee M.A., Pratt H., Zheng Y. (2018). Dense Fully Convolutional Segmentation of the Optic Disc and Cup in Colour Fundus for Glaucoma Diagnosis. Symmetry.

[20] Zilly J., Buhmann J.M., Mahapatra D. (2017). Glaucoma Detection Using Entropy Sampling and Ensemble Learning for Automatic Optic Cup and Disc Segmentation. Comput. Med. Imaging Graph..

[21] Liu H., Li L., Wormstone I.M., Qiao C., Zhang C., Liu P., Li S., Wang H., Mou D., Pang R. (2019). Development and Validation of a Deep Learning System to Detect Glaucomatous Optic Neuropathy Using Fundus Photographs. JAMA Ophthalmol..

[22] Shibata N., Tanito M., Mitsuhashi K., Fujino Y., Matsuura M., Murata H., Asaoka R. (2018). Development of a Deep Residual Learning Algorithm to Screen for Glaucoma from Fundus Photography. Sci. Rep..

[23] Ting D.S.W., Cheung C.Y.-L., Lim G., Tan G.S.W., Quang N.D., Gan A., Hamzah H., Garcia-Franco R., San Yeo I.Y., Lee S.Y. (2017). Development and Validation of a Deep Learning System for Diabetic Retinopathy and Related Eye Diseases Using Retinal Images From Multiethnic Populations With Diabetes. JAMA.

[24] Barman S.A., Welikala R.A., Rudnicka A.R., Owen C.G., Trucco E., MacGillivray T., Xu Y. (2019). Chapter 8—Image Quality Assessment. Computational Retinal Image Analysis.

[25] Fu H., Wang B., Shen J., Cui S., Xu Y., Liu J., Shao L. Evaluation of Retinal Image Quality Assessment Networks in Different Color-Spaces. Proceedings of the 22nd International Conference on Medical Image Computing and Computer-Assisted Intervention, MICCAI 2019.

[26] Fatima K.N., Hassan T., Akram M.U., Akhtar M., Butt W.H. (2017). Fully Automated Diagnosis of Papilledema through Robust Extraction of Vascular Patterns and Ocular Pathology from Fundus Photographs. Biomed. Opt. Express.

[27] Saha S.K., Fernando B., Cuadros J., Xiao D., Kanagasingam Y. (2018). Automated Quality Assessment of Colour Fundus Images for Diabetic Retinopathy Screening in Telemedicine. J. Digit. Imaging.

[28] Zago G.T., Andreão R.V., Dorizzi B., Teatini Salles E.O. (2018). Retinal Image Quality Assessment Using Deep Learning. Comput. Biol. Med..

[29] Chalakkal R.J., Abdulla W.H., Thulaseedharan S.S. (2019). Quality and Content Analysis of Fundus Images Using Deep Learning. Comput. Biol. Med..

[30] Xiong L., Li H. (2016). An Approach to Locate Optic Disc in Retinal Images with Pathological Changes. Comput. Med. Imaging Graph..

[31] Li Z., He Y., Keel S., Meng W., Chang R.T., He M. (2018). Efficacy of a Deep Learning System for Detecting Glaucomatous Optic Neuropathy Based on Color Fundus Photographs. Ophthalmology.

[32] Giancardo L., Meriaudeau F., Karnowski T.P., Chaum E., Tobin K., Campolo D. (2010). Quality Assessment of Retinal Fundus Images Using Elliptical Local Vessel Density. New Developments in Biomedical Engineering.

[33] Fleming A.D., Philip S., Goatman K.A., Olson J.A., Sharp P.F. (2006). Automated Assessment of Diabetic Retinal Image Quality Based on Clarity and Field Definition. Investig. Ophthalmol. Vis. Sci..

[34] Khan S.M., Liu X., Nath S., Korot E., Faes L., Wagner S.K., Keane P.A., Sebire N.J., Burton M.J., Denniston A.K. (2021). A Global Review of Publicly Available Datasets for Ophthalmological Imaging: Barriers to Access, Usability, and Generalisability. Lancet Digit. Health.

[35] Mahapatra D., Roy P.K., Sedai S., Garnavi R. A CNN Based Neurobiology Inspired Approach for Retinal Image Quality Assessment. Proceedings of the 2016 38th Annual International Conference of the IEEE Engineering in Medicine and Biology Society (EMBC).

[36] Zapata M.A., Royo-Fibla D., Font O., Vela J.I., Marcantonio I., Moya-Sánchez E.U., Sánchez-Pérez A., Garcia-Gasulla D., Cortés U., Ayguadé E. (2020). Artificial Intelligence to Identify Retinal Fundus Images, Quality Validation, Laterality Evaluation, Macular Degeneration, and Suspected Glaucoma. Clin. Ophthalmol. Auckl. NZ.

[37] Bhatkalkar B., Joshi A., Prabhu S., Bhandary S. (2020). Automated Fundus Image Quality Assessment and Segmentation of Optic Disc Using Convolutional Neural Networks. Int. J. Electr. Comput. Eng. IJECE.

[38] Korot E., Guan Z., Ferraz D., Wagner S.K., Zhang G., Liu X., Faes L., Pontikos N., Finlayson S.G., Khalid H. (2021). Code-Free Deep Learning for Multi-Modality Medical Image Classification. Nat. Mach. Intell..

[39] Wang X., Zhang S., Liang X., Zheng C., Zheng J., Sun M. (2019). A Cnn-Based Retinal Image Quality Assessment System for Teleophthalmology. J. Mech. Med. Biol..

